# Pre‐drought Conservative Xylem Hydraulic Architecture Is Associated With Enhanced Recovery From the 1976 Drought in European Beech (*Fagus sylvatica* L.)

**DOI:** 10.1111/pce.70691

**Published:** 2026-06-21

**Authors:** Simon Röder, Edurne Martinez del Castillo, Max Torbenson, Heike Zimmer‐Zachmann, Frederick Reinig, Antonia Kölzer, Ulf Büntgen, Jan Esper, Emanuele Ziaco

**Affiliations:** ^1^ Department of Geography Johannes Gutenberg University Mainz Germany; ^2^ Department of Geography Texas A&M University College Station Texas USA; ^3^ Department of Geography University of Cambridge Cambridge UK; ^4^ Global Change Research Institute of the Czech Academy of Sciences Brno Czech Republic; ^5^ Department of Geography Faculty of Science Masaryk University Brno Czech Republic

## Abstract

Beech (*Fagus sylvatica* L.) trees with safer xylem hydraulic architecture recovered better from the 1976 drought than hydraulically efficient individuals, showing that conservative xylem structures, rather than high growth rates, may support resilience to extreme drought.

Droughts are expected to increase in frequency and intensity under future climate change, threatening the capacity of temperate forests to preserve ecosystem services. Basal area increment (BAI) is widely used as a proxy of secondary growth to quantify species resilience to drought at both the tree and ecosystem levels. It is often assumed that vigorous trees with high pre‐drought growth rates exhibit fast post‐drought recovery, while slow‐growing trees are commonly associated with conservative strategies enhancing drought resistance (Cailleret et al. 2017). However, a process‐based understanding of the underlying mechanisms linking BAI to drought resilience in temperate forests is far from complete. In this regard, wood cellular features such as vessel size, density and lumen area have long been interpreted as key determinants of xylem hydraulic strategies and ecological adaptation to environmental stress (Carlquist 2001).

In recent years, beech (*Fagus sylvatica* L.) populations have experienced widespread mortality across their distribution range (Leuschner 2020). Historical droughts offer opportunities to investigate how xylem hydraulic structure influence long‐term legacies of extreme events on post‐drought growth patterns. Here we studied an even‐aged, managed beech stand in central Germany (Supporting Information: Figure S1), to investigate growth responses and wood anatomical traits before and after the exceptional drought of 1976 (Supporting Information: Figure S2). For detailed quantitative wood anatomy (QWA) analysis (Figure 1a) we selected two sub‐groups of trees showing striking divergence in post‐drought growth patterns (Figure 1b): one characterised by a rapid recovery (FAST, 4 trees) and another by prolonged growth suppression (SLOW, 4 trees), with similar mean age (respectively 86 and 78 years) (Supporting Information: Figure S3).

**Figure 1:**
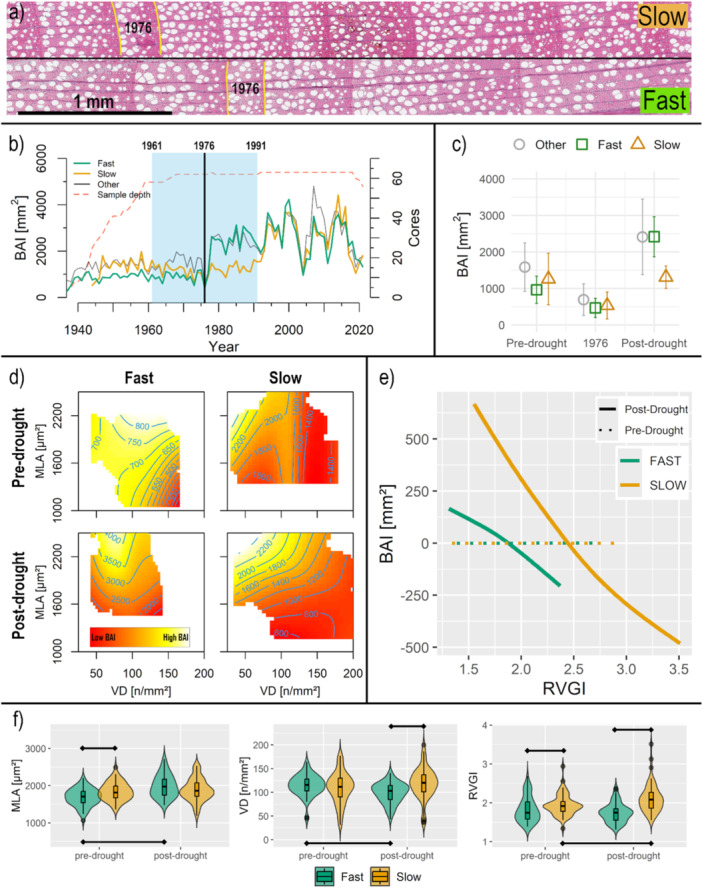
(a) Wood anatomical sections (magnification 40 x) of a SLOW and FAST tree (the 1976 rings are marked in yellow); (b) mean BAI chronologies for FAST and SLOW trees compared to the mean site BAI chronology derived from the remaining sampled trees (Other). Blue shaded area indicates the 15‐year pre‐ and post‐ 1976 drought periods; (c) BAI for FAST, SLOW and OTHER trees before, during and after the 1976 drought (symbols are group means ± 1 SD); (d) 2D surfaces showing the effect of GAMM‐estimated interaction between vessel density (VD) and mean lumen area (MLA) on BAI in the pre‐ (upper panels) and post‐drought (lower panels) periods for FAST (left panels) and SLOW trees (right panels). White areas indicate regions of the predictor space not covered by observations; (e) GAMM‐estimated partial effect of vessel grouping index (RVGI) on BAI for FAST (green lines) and SLOW trees (orange lines) modelled separately for the pre‐ (dotted lines) and post‐1976 (solid lines) drought periods. The *y*‐axis represents the modelled contribution of RVGI to BAI (not observed BAI); (f) violin plots showing pre‐ and post‐1976 distribution of MLA, VD and RVGI. Boxplots indicate the interquartile range and median. Horizontal lines highlight statistically significant differences (*p* < 0.05) between groups according to the Wilcoxon sum‐rank test.

In terms of secondary growth, the 1976 drought had a slightly higher impact on the SLOW trees, causing a 57.6% reduction in BAI compared to the pre‐drought period (1961–1975); in contrast, the FAST trees experienced a decrease of 51.5% while the mean BAI reduction for the rest of the sampled trees (‘OTHER’) was 56.3% (Figure 1c). Post‐drought (1977–1991) growth rates in FAST nearly doubled (2415 ± 548 mm^2^/year) those of SLOW (1307 ± 307 mm^2^/year). Interestingly, during the pre‐drought period, FAST exhibited much lower growth rates (961 ± 373 mm^2^/year) than SLOW (1259 ± 705 mm^2^/year). Overall, mean post‐drought BAI in the FAST group increased by + 151% compared to pre‐drought levels, whereas in SLOW, BAI remained unchanged (+ 0.4%), given a + 52% increase at the population level.

BAI was modelled as a function of xylem functional and cellular traits (Supporting Information: Figure S4 and Table S1) using generalised additive mixed models (GAMMs) (Supplementary Methods). The best‐fitting general model for all trees pooled together across the whole study period (1944–2021) included the interaction between vessel density (VD) and mean lumen area (MLA) as the primary driver of BAI (*F* = 92.438, *p* < 0.001) (Supporting Information: Figure S5 and Table S2), with ring vessel grouping index (RVGI) showing a weaker but significant effect. However, the general model explained a smaller proportion of variance in BAI (*R* ^2^_adj_ = 0.436; Supporting Information: Table S2) compared with models fitted separately for FAST and SLOW trees, which indicated differences in the relationship between xylem traits and BAI across the two groups (Figure 1d). In the pre‐drought period, the interacting factor VD × MLA played a primary role in determining BAI, but the effect was stronger in FAST (*F* = 9.027, *p* = 0.006) than SLOW trees (*F* = 4.438, *p* = 0.0017) while RVGI was insignificant in both groups (Figure 1e and Supporting Information: Table S2). The random factor tree accounted for a large portion of the observed variability, particularly in FAST (*F* = 43.182 vs. *F* = 7.475 in SLOW).

In the post‐drought period, the contribution of wood anatomical predictors to BAI changed markedly. In the FAST‐recovering trees, the VD × MLA interaction became much stronger (*F* = 44.969, *p* < 0.001), while the contribution of RVGI remained nonsignificant. Conversely, in SLOW, RVGI became a predictor of BAI (*F* = 1.352, *p* < 0.001). Such behaviour was more uniform across the SLOW trees, as individual variability was no longer significant after the drought. Model fit improved for both groups (Supporting Information: Table S2); however, post‐drought mechanisms of growth recovery differed, with FAST becoming more dependent on the MLA × VD trade‐off and SLOW showing a higher impact of RVGI on BAI (Figure 1e).

The variability of MLA, VD and RVGI pre‐ and post‐drought clearly demonstrated that before 1976 the SLOW trees exhibited vessel characteristics consistent with high hydraulic efficiency, with larger MLA and lower VD, which supported their sustained growth rates (Figure 1f and Supporting Information: Table S3). In contrast, FAST outlined conservative xylem traits, with smaller vessels and higher density, reflecting a safety‐oriented strategy. After the drought, FAST showed marked plasticity, with MLA increasing by + 17.0%, while VD declined by − 11.5%. The SLOW trees, in turn, exhibited limited anatomical adjustments: most traits showed only moderate changes, with the main shift being an increase in VD (+ 10.6%) and RVGI (+ 8.3%), a configuration associated with higher hydraulic safety (Figure 1f).

These findings reveal that high pre‐drought growth rates are not necessarily a predisposing factor for resilience. Recent evidence shows that drought resilience reflects system‐level hydraulic organisation and redundancy, which shape plant responses and recovery under water stress (Yao et al. 2025). Here, we show that sustained growth rates are associated with efficient xylem hydraulic structures, which increase water conductivity. In contrast, slow‐growing trees tend to exhibit more conservative xylem traits, reducing conductivity (Schuldt et al. 2016). The SLOW trees were likely dominant in 1976, exposed to greater evaporative demand and therefore more vulnerable to water limitations. These individuals likely experienced sustained xylem and canopy damage, which caused them to remain competitively disadvantaged for decades thereafter. On the other hand, the FAST trees, which showed low pre‐drought growth likely due to unfavourable canopy positions, were able to capitalise on their safer xylem hydraulic architecture to limit xylem and canopy damages during the drought, while quickly responding once competition was relaxed by canopy decline in neighbouring trees.

Our conclusions are based on retrospective observations and limited to a single forest stand. Yet, these dynamics appear to be consistent with the intense size‐symmetric competition typical of beech forests in the early stages of their structural development, and supported by the homogeneous site conditions and forest structure. Under such conditions, beech is highly sensitive to canopy perturbations, which are quickly recorded in tree rings and their cellular structure. Moreover, on a flat, homogeneous, relatively small forest parcel, microclimatic or edaphic differences are unlikely to explain the observed variability. On the contrary, they suggest that severe droughts can act as a selective disturbance reshuffling competitive hierarchies (Cavin et al. 2013), challenging the common view that vigour equals resilience and emphasising the ecological importance of xylem safety in coping with extreme events.

Our study highlights the potential of QWA as a tool to identify predisposing xylem traits for withstanding extreme droughts. Our results are consistent with the long‐standing view that xylem cellular organisation reflects functionally relevant hydraulic adjustments (Carlquist 2001). However, since QWA is based on retrospectively quantifiable xylem anatomical traits, our inferences are restricted to tree xylem hydraulic organisation as measurable at breast height, and its long‐term functional consequences, rather than assessing how pit‐level embolism resistance thresholds or transient physiological states such as native embolism and instantaneous hydraulic failure control drought resilience (Peters and Choat 2025). Yet, the patterns we observed agree with broader evidence that xylem hydraulic structure is a critical determinant of drought survival in beech (Walthert et al. 2021). Other angiosperms have been shown to modulate vessel size, density or grouping in response to water stress. We extend this understanding by demonstrating that within a single species and stand, contrasting xylem hydraulic architectures are associated with radically different long‐term outcomes after drought (Gessler et al. 2020). Resilience manifested as either the opportunistic release of individuals with limited access to the canopy or the conservative persistence of dominants at lower growth. This diversity of pathways underscores the importance of maintaining structural and functional heterogeneity within stands.

From a management perspective, these results have significant implications. In even‐aged beech forests, competitively disadvantaged individuals are often removed in thinning regimes, yet they may constitute a latent reservoir of resilience. This mechanism resembles a form of demographic insurance, as such trees with safer hydraulics may persist through stressful periods and rebound when canopy openings occur. Silvicultural practices that favour uniformity and eliminate suppressed trees may reduce stand‐level resilience by eroding this hidden potential (Meyer et al. 2022).

The 1976 drought reveals how extreme climate events can override assumptions about vigour and resilience, showing that slow predrought growth does not necessarily indicate susceptibility but may conceal a latent capacity for recovery rooted in hydraulic safety. High growth is no guarantee of resilience but may predispose individuals to long‐term suppression if efficient xylem fails during stress. By linking growth trajectories with QWA, we were able to disentangle these strategies and highlight the ecological value of conservative hydraulic design. Diversity in xylem functional traits among coexisting species likely buffer forest communities against drought impacts and contribute to greater overall resistance (Langan et al. 2025). As droughts intensify, recognising and preserving the diversity of hydraulic traits for drought resilience could prove essential to sustaining forest functioning and productivity.

## Conflicts of Interest

The authors declare no conflicts of interest.

## Supporting information

Supporting File

## Data Availability

The data that support the findings of this study are available from the corresponding author upon request.
