# Supplementary material for: Pre‐drought Conservative Xylem Hydraulic Architecture Is Associated With Enhanced Recovery From the 1976 Drought in European Beech (Fagus sylvatica L.)
Source: Plant Cell Environ. 2026 Jun 21;49(9):7215–8. doi: 10.1111/pce.70691 (PMC13436499; doi:10.1111/pce.70691)
Supplement: Supplementary file 1 [file PCE-49-7215-s001.docx]

***Brief communication***

**Pre-drought conservative xylem hydraulic architecture is associated with enhanced recovery from the 1976 drought in European beech (*Fagus sylvatica* L.)**

Simon Röder^1^, Edurne Martinez del Castillo^1^, Max Torbenson^1,2^, Heike Zimmer-Zachmann^1^, Frederick Reinig^1^, Antonia Kölzer^1^, Ulf Büntgen^3,4,5^, Jan Esper^1,4^, Emanuele Ziaco^1*^

*^1^ Department of Geography, Johannes Gutenberg University, 55099 Mainz, Germany*

*^2^ Department of Geography, Texas A&M University, 77843 College Station, TX, USA*

*^3^ Department of Geography, University of Cambridge, Cambridge CB2 3EN, UK*

*^4^ Global Change Research Institute of the Czech Academy of Sciences, 60300 Brno, Czech Republic*

*^5^ Department of Geography, Faculty of Science, Masaryk University, 61137 Brno, Czech Republic*

*Corresponding author: [eziaco@uni-mainz.de](mailto:eziaco@uni-mainz.de)

**SUPPLEMENTARY METHODS**

**1.1 | Study area and the 1976 drought**

The study was conducted in a managed mixed forest composed predominantly of European beech (*Fagus sylvatica*) and Scots pine (*Pinus sylvestris* L.) located in the Hessian Rhine Plain (Germany) (49.96° N, 8.52° E; 110 m a.s.l.) (Figure S1). The site is situated on flat terrain, characterized by deep, carbonate-rich clay and sandy soil, which ensures a medium- to good nutrient supply (HessenForst 2019). Local climate is characterized by warm summers and mild winters, with a mean annual temperature of 10.0 °C and annual precipitation totals of 685 mm from 1920 to 2022 (Copernicus E-OBS) (Haylock et al. 2008).

This region was heavily impacted by the extreme summer drought of 1976, one of the worst in central Europe over the past 2,000 years (Torbenson et al. 2023), that triggered widespread forest decline and mortality (Schütt and Cowling 1985). At the continental level, the 1976 drought started in May and continued throughout the entire summer (Spinoni et al. 2015). At the study area, the 1976 drought was the most severe one in the 20th century, according to the Standardized Precipitation-Evapotranspiration Index (SPEI) (Beguería et al. 2014). It peaked in June, with the 1976 June SPEI2 (-2.6) ranking as the lowest on records for the period 1901-2024 (Figure S2). Other significant events were the 2003 drought, with a September SPEI6 of -1.9 (*data not shown*), and the recent drought of 2023, with a June SPEI2 of -2.5.

**1.2 | Sampling design**

In August 2022, a total of 64 cores were collected at breast height (1,3 m from the ground) from 32 beech trees (two cores per tree from opposite directions) using a 5-mm increment borer (Figure S3). The average diameter of sampled trees at breast height (DBH) was 46 ± 10 cm. After sampling, cores were glued to wooden holders with water-soluble wood glue and air dried. Samples were surfaced using sandpaper with progressively finer grit to enhance the visibility of the annual rings. Tree ring-width (TRW) was measured with an accuracy of 0.01 mm using a LINTAB 6 measuring device and the task-specific software TSAP (Rinntech-Metriwerk GmbH & Co.KG, Heidelberg, Germany). Samples were crossdated using Cofecha (Holmes 1983) to develop an annually resolved site chronology. Individual TRW series were then converted into basal area increment (BAI) series (Biondi and Qeadan 2008) using the function *bai.out()* in the R package *dplR* (Bunn et al. 2014). TRW and BAI series were finally averaged to a mean site chronology using Tukey’s biweight robust mean.

All trees included in this study are healthy, even-aged, dominant individuals showing no signs of growth decline or canopy dieback after recent droughts (i.e. 2018-2019). However, a preliminary analysis of the TRW series revealed high variability in post-drought recovery patterns following the drought of 1976. The 1976 drought was chosen in our study as it represented the first extreme drought experienced by these trees, occurring at a time when the stand still exhibited a developing structure with intense competition among individuals, as indicated by dendrochronological evidence. Under these conditions, we could investigate the impact of drought on tree growth and xylem anatomy without any legacy effects from previous events. Hence, for quantitative wood anatomical analyses we selected a subset of eight trees, four of which demonstrated exceptionally fast post-drought recovery (hereinafter referred to as “FAST”) and four exhibited slow-recovery (referred to as “SLOW”) in response to the 1976 drought. FAST and SLOW trees were not pre-selected based on social position or growth status at the time of sampling, but were identified *a posteriori* based on their contrasting post-drought recovery dynamics following the 1976 event. The FAST trees showed above-average TRW and BAI until the mid-1990s, whereas the SLOW trees had consistently lower-than-average growth rates during approximately the same timeframe. Site characteristics and forest structure provided suitable conditions to assess the effect of xylem cellular structure on post-drought recovery trajectories. Trees were spatially clustered within a very small area: the area enclosing all trees was ~3,000 m² (~0.30 ha), with maximum site extents of ~75 m (E–W) and ~55 m (N–S), and the mean distance among sampled individuals was 32.1 m (maximum distance 76.4 m) (Figure S1). The similar age and size of these trees, their proximity, and the flat topography of the study site, suggest that growing conditions can be considered close to identical so that age-related or microsite effects (i.e. differences in soil moisture) may be excluded.

**1.3 | Quantitative wood anatomical measurements**

Following standard procedure, one core from each FAST and SLOW tree was processed for QWA analysis (von Arx et al., 2016). Cores were cut diagonally into 3 - 4 cm long pieces, to maintain overlapping rings across consecutive pieces, and each piece was boiled in distilled water for 15 minutes. Ten-twelve µm thick micro sections were obtained using a rotary microtome (Leica, Heidelberg,Germany) and bleached in 10% sodium hypochlorite for 10 minutes to decolorize cell walls. Finally, the samples were rinsed in water and microsections were stained in a 1:1 Safranin-Astrablue solution, dehydrated in successive ethanol baths (50% and 100% pure), permanently mounted on microscopic slides with EUKITT™ (ORSAtec, Bobingen, Germany), and dried in the oven at 60°C for 48 hours.

High-resolution digital images with 20x magnification were obtained with a NanoZoomer 2.0-HAT slide scanner (HAMAMATSU PHOTONICS Europe GmbH, Herrsching, Germany). This resulted in 225 digital images for which quantitative anatomical parameters were acquired through the software ROXAS (von Arx and Carrer 2014). Measured xylem vessel features included: vessel number (VNo), vessel density (VD), relative conductive area (RCTA), mean vessel lumen area (MLA), maximum vessel lumen area (MaxLA), theoretical xylem specific hydraulic conductivity (Ks), vessel grouping index (RVGI), vessel solitary fraction (RVSF) and mean hydraulic diameter (Dh) (Table S1). All anatomical and functional traits were finally averaged in a mean site chronology and in a separate chronology for the FAST and SLOW recovering trees. Wood anatomical chronologies covered the period 1938-2021 for FAST and 1946-2021 for SLOW recovering trees.

**1.4 | Statistical analysis**

To investigate changes in beech wood anatomical structure in response to the 1976 drought, all chronologies were examined for a 15-years pre- (1961-1975) and post-event periods (1977-1991). The 15-year post-drought period corresponded to the average time needed for SLOW recovering trees to reach the stand’s average growth rates. Additionally, a period of 15 years was deemed adequate to provide a more accurate description of baseline pre-drought conditions, considering that in the diffuse-porous xylem structure of *F. sylvatica* L., vessels from previous years contribute to water transport in the following ones (Gasson 1985). A Shapiro-Wilk normality test was applied to the distribution of growth and xylem functional traits. Since our data were not normally distributed, we used the Wilcoxon rank-sum test to check differences in the pre- and post-drought median values and directional shifts in the distribution of xylem anatomical and functional traits.

BAI was modeled as a function of wood anatomical traits using generalized additive mixed models (GAMMs), which allow for non‐linear relationships (Wood 2017). Prior to model fitting, we assessed multicollinearity among predictors, by computing distance correlations among all anatomical and functional traits. Predictors with high linear and non‐linear dependencies (|distance correlation| > 0.7) were identified and excluded from subsequent analyses to ensure that only the most informative variables were retained, using the *caret* package (Kuhn 2008). First, we tested several GAMMs across the entire period 1944-2021, combining FAST and SLOW trees, to determine the best predictors of BAI regardless of the response to the 1976 drought. The final GAMM included MLA, VD, and RVGI as fixed effects and trees as random factors, and was specified as follows:

*BAI* ~ *te*(*VD*, *MLA*) + *s*(*RVGI*) + *s*(*tree*)

where *te*(*VD*,*MLA*) is a tensor product smooth capturing the interaction between vessel density and mean lumen area, *s(RVGI)* is a univariate smooth term for vessel grouping index, and *s(tree)* represents the random effect for tree identity. The model was fitted using restricted maximum likelihood (REML) in the R package *mgcv* (Wood, 2017). The model was then fitted separately to the FAST and SLOW trees in the pre- (1961-1975) and post-drought (1977-1991) periods, to evaluate potential shifts in the contribution of each parameter to BAI. The interacting effects of VD-MLA and the independent impact of RVGI were extracted and visualized to identify changes in anatomy-growth relationships between pre- and post-drought.

**References**

Beguería, S., S.M.V. Serrano, F. Reig-Gracia, et al. 2014. Standardized precipitation evapotranspiration index (SPEI) revisited: parameter fitting, evapotranspiration models, tools, datasets and drought monitoring. Wiley-Blackwell.

Biondi, F. and F. Qeadan. 2008. A Theory-Driven Approach to Tree-Ring Standardization: Defining the Biological Trend from Expected Basal Area Increment. Tree-Ring Research. 64:81-96, 16.

Bunn, A.G., M. Korpela, F. Biondi, et al. 2014. dplR: Dendrochronology Program Library in R. <http://CRAN.R-project.org/package=dplR>.

Gasson, P. 1985. Automatic Measurement of Vessel Lumen Area and Diameter With Particular Reference to Pedunculate Oak and Common Beech. IAWA Journal. 6:219-237.

Haylock, M.R., N. Hofstra, A.M.G. Klein Tank, et al. 2008. A European daily high-resolution gridded data set of surface temperature and precipitation for 1950–2006. Journal of Geophysical Research: Atmospheres. 113

HessenForst. 2019. Landesbetrieb HessenForst. 1129-1-130-171 Staatswald FA Groß-Gerau, Revier: Hassloch.

Holmes, R.L. 1983. Computer-Assisted Quality Control in Tree-Ring Dating and Measurement. Tree-ring Bulletin

Kuhn, M. 2008. Building Predictive Models in R Using the caret Package. Journal of Statistical Software. 28:1 - 26.

Schütt, P. and E. Cowling. 1985. Waldsterben, a general decline of forests in central Europe: symptoms, development and possible causes. Plant Disease. 69:548–558.

Spinoni, J., G. Naumann, J.V. Vogt, et al. 2015. The biggest drought events in Europe from 1950 to 2012. Journal of Hydrology: Regional Studies. 3:509-524.

Torbenson, M.C.A., U. Büntgen, J. Esper, et al. 2023. Central European Agroclimate over the Past 2000 Years. Journal of Climate. 36:4429-4441.

von Arx, G. and M. Carrer. 2014. ROXAS – A new tool to build centuries-long tracheid-lumen chronologies in conifers. Dendrochronologia. 32:290-293.

Wood, S.N. 2017. Generalized Additive Models: An Introduction with R, Second Edition (2nd ed.). Chapman and Hall/CRC.

**SUPPLEMENTARY TABLES**

**Table S1** Description of wood anatomical and functional traits measured in this study.

| **Parameter** | **Acronym** | **Unit** | **Description** |
| --- | --- | --- | --- |
| Vessel Number | VNo | No. | Total number of vessels |
| Vessel Density | VD | No./mm^2^ | Number of vessels per unit area |
| Mean Vessel Area | MLA | µm^2^ | Mean cross-sectional vessel lumen area |
| Maximum Vessel Area | MaxLA | µm^2^ | Vessel cross-sectional lumen area of the largest vessel |
| Relative Conductive Area | RCTA | % | Percentage of total conductive area over total ring area |
| Xylem Specific Conductivity | Ks | m^2^s^-1^MPa^-1^ | Theoretical xylem hydraulic conductivity per unit area |
| Hydraulic Diameter | Dh | µm | Mean hydraulic diameter of vessels per ring |
| Vessel Grouping Index | RVGI | - | Vessel Grouping Index: Mean number of vessels per group per annual ring |
| Vessel Solitary Fraction | RVSF | % | fraction of solitary vessels with respect to all vessels in a ring |

**Table S2** Summary of Generalized Additive Mixed Models (GAMMs) describing anatomy-BAI relationships during different periods (full, pre-drought, post-drought) and tree groups (FAST vs. SLOW). The table reports effective degrees of freedom (edf), F-values, and p-values for each smooth term, along with model deviance explained, adjusted R², and number of observations (*n*) (** p < 0.05; **p < 0.01; ***p < 0.001*).

| **Full model (1944-2021)** | | | |
| --- | --- | --- | --- |
| Smooth term | Edf | F-value | p-value |
| te(VD, MLA) | 9.590 | 92.438 | < 0.001*** |
| s(RVGI) | 1.264 | 1.357 | < 0.01** |
| s(tree) | 6.519 | 14.731 | < 0.001*** |
| Deviance explained | 45.2% |  |  |
| R^2^_adj_ | 0.43 |  |  |
| Observation (n) | 599 |  |  |
| **FAST pre-drought (1961-1975)** | | | |
| Smooth term | Edf | F-value | p-value |
| te(VD, MLA) | 2.665 | 9.027 | < 0.01** |
| s(RVGI) | 0.006 | 0.001 | 0.321 |
| s(tree) | 2.939 | 43.182 | < 0.001*** |
| Deviance explained | 74.5% |  |  |
| R^2^_adj_ | 0.71 |  |  |
| Observation (n) | 60 |  |  |
| **SLOW pre-drought (1961-1975)** | | | |
| Smooth term | Edf | F-value | p-value |
| te(VD, MLA) | 4.373 | 4.438 | < 0.01** |
| s(RVGI) | 0.000 | 0.000 | 0.546 |
| s(tree) | 2.622 | 7.475 | < 0.001*** |
| Deviance explained | 61.2% |  |  |
| R^2^_adj_ | 0.53 |  |  |
| Observation (n) | 60 |  |  |
| **FAST post-drought (1977-1991)** | | | |
| Smooth term | Edf | F-value | p-value |
| te(VD, MLA) | 7.606 | 44.969 | < 0.001*** |
| s(RVGI) | 0.695 | 0.256 | 0.134 |
| s(tree) | 2.881 | 26.328 | < 0.001*** |
| Deviance explained | 77.0% |  |  |
| R^2^_adj_ | 0.70 |  |  |
| Observation (n) | 60 |  |  |
| **SLOW post-drought (1977-1991)** | | | |
| Smooth term | Edf | F-value | p-value |
| te(VD, MLA) | 5.621 | 2.802 | < 0.001*** |
| s(RVGI) | 1.514 | 1.352 | < 0.001*** |
| s(tree) | 0.001 | 0.000 | 0.592 |
| Deviance explained | 66.5% |  |  |
| R^2^_adj_ | 0.60 |  |  |
| Observation (n) | 60 |  |  |

**Table S3** Summary of mean pre- and post-drought anatomical parameters, relative differences between post- and pre-drought (Δ_post-pre_), SLOW and FAST groups (Δ_slow-fast_) mean values, and Wilcoxon Sum-Rank test statistics for the medians between and within groups and periods (*** *p < 0.001*; ** *p < 0.01*; * *p < 0.05*; ^+^ *n.s.*)

| **Parameter** | **Unit** | **Group** | **Pre-drought**  **(1961-1975)** | **Post-drought**  **(1977-1991)** | **Δ_post-pre_** | **W_post-pre_** |
| --- | --- | --- | --- | --- | --- | --- |
| Vno | No. | FAST | 363 | 535 | +47.2% | 948*** |
|  |  | SLOW | 337 | 350 | +4.0% | 1784^+^ |
|  |  | Δ_slow-fast_ | -7.3% | -34.5% |  |  |
|  |  | W_slow-fast_ | 1913^+^ | 2798*** |  |  |
| VD | No./mm^2^ | FAST | 115 | 102 | -11.5% | 2535*** |
|  |  | SLOW | 110 | 121 | +10.6% | 1492^+^ |
|  |  | Δ_slow-fast_ | -4.8% | +19.1% |  |  |
|  |  | W_slow-fast_ | 1962^+^ | 1001*** |  |  |
| MLA | µm^2^ | FAST | 1678 | 1963 | +17.0% | 858*** |
|  |  | SLOW | 1837 | 1880 | +2.3% | 1712^+^ |
|  |  | Δ_slow-fast_ | +9.5% | -4.2% |  |  |
|  |  | W_slow-fast_ | 1180** | 2092^+^ |  |  |
| MaxLA | µm^2^ | FAST | 4474 | 5455 | +21.9% | 852*** |
|  |  | SLOW | 4974 | 4742 | -4.7% | 2048^+^ |
|  |  | Δ_slow-fast_ | +11.2% | -13.0% |  |  |
|  |  | W_slow-fast_ | 1193** | 2409** |  |  |
| RCTA | % | FAST | 19.5 | 19.2 | -1.6% | 1798^+^ |
|  |  | SLOW | 21.1 | 22.4 | +6.3% | 1496^+^ |
|  |  | Δ_slow-fast_ | +8.0% | +16.6% |  |  |
|  |  | W_slow-fast_ | 1381* | 1055*** |  |  |
| Ks | m^2^s^-1^MPa^-1^ | FAST | 0.0153 | 0.0181 | +18.3% | 1256** |
|  |  | SLOW | 0.0176 | 0.0191 | +8.3% | 1562^+^ |
|  |  | Δ_slow-fast_ | +15.9% | +6.0% |  |  |
|  |  | W_slow-fast_ | 1382* | 1577^+^ |  |  |
| Dh | µm | FAST | 48.3 | 52.1 | +7.8% | 935*** |
|  |  | SLOW | 50.7 | 50.8 | +0.2% | 1754^+^ |
|  |  | Δ_slow-fast_ | +4.8% | -2.5% |  |  |
|  |  | W_slow-fast_ | 1196** | 2108^+^ |  |  |
| RVGI | - | FAST | 1.80 | 1.75 | -3.0% | 1998^+^ |
|  |  | SLOW | 1.92 | 2.08 | +8.3% | 1272** |
|  |  | Δ_slow-fast_ | +6.8% | +19.1% |  |  |
|  |  | W_slow-fast_ | 1324* | 659*** |  |  |
| RVSF | % | FAST | 35.3 | 37.2 | +5.4% | 1500^+^ |
|  |  | SLOW | 30.4 | 27.8 | -8.7% | 2212* |
|  |  | Δ_slow-fast_ | -13.9% | -25.4% |  |  |
|  |  | W_slow-fast_ | 2325** | 2994*** |  |  |

**SUPPLEMENTARY FIGURES**

**Figure S1** a) Location of the study site in central-western Germany; b) distribution of sampled trees: FAST (green symbols) and SLOW recovering trees (red symbols). Other trees included in the TRW and BAI analysis are shown as white symbols.

**Figure S2** Temporal variation of June SPEI2 for the period 1901-2024 in the study area. Yellow diamond highlights the 1976 drought. Horizontal dashed blue lines are ± 2 standard deviations from the long-term mean (1901-2024; horizontal solid blue line). Yellow line is the 11-yrs running mean for June SPEI2.

**Figure S3** Segment plot showing the lifespan of all sampled trees, starting with the year of the oldest crossdated ring in the two cores extracted from each tree, and ending with the year 2021 (the date of the last crossdated ring). Green and orange bars represent respectively FAST and SLOW trees included in the analysis.

**Figure S4** Mean chronologies of wood anatomical parameters (see Table S1 for acronyms) for FAST (green curves) and SLOW recovering trees (orange). Blue shaded areas indicate the 15-year periods prior and after the 1976 drought.

**Figure S5** Contour plot showing the generalized additive mixed model (GAMM)-estimated interaction between vessel density (VD) and mean lumen area (MLA) on basal area increment (BAI) on all FAST- and SLOW-growing trees combined in the period 1944-2021. Contour lines represent estimated BAI values, with yellow areas indicating higher predicted BAI, and red ones indicating lower values.
